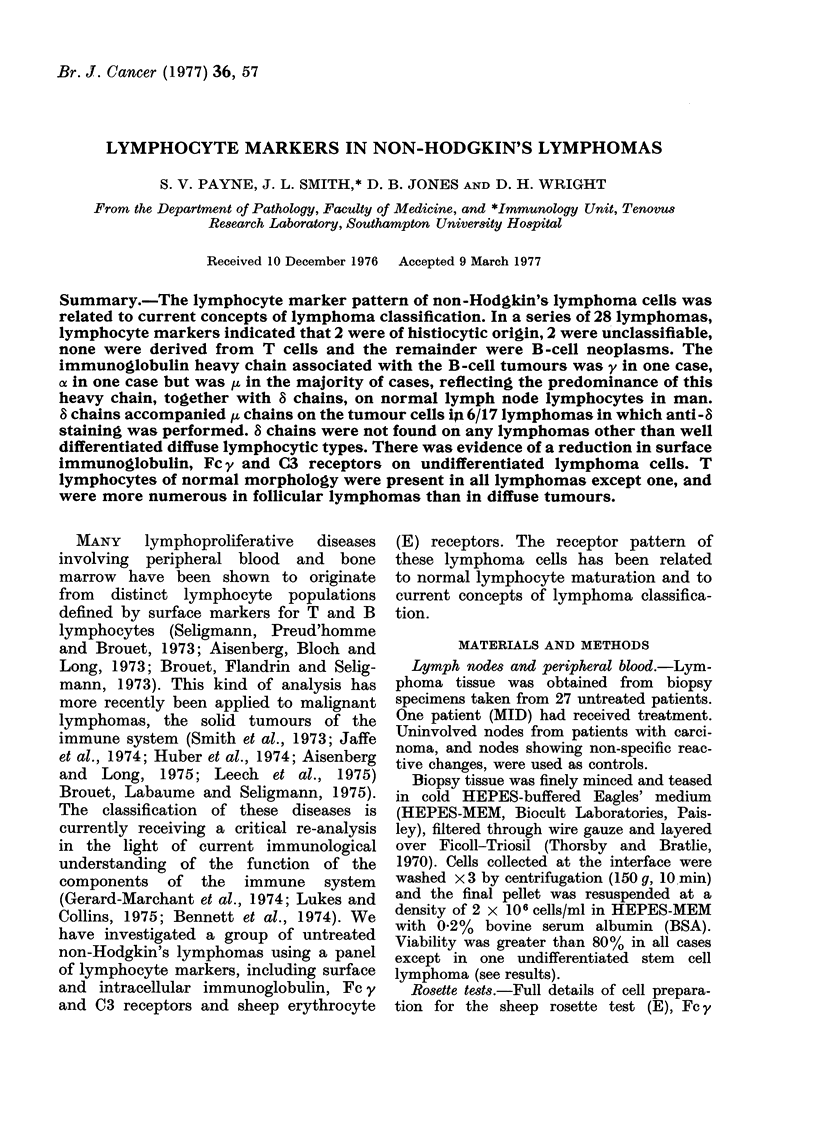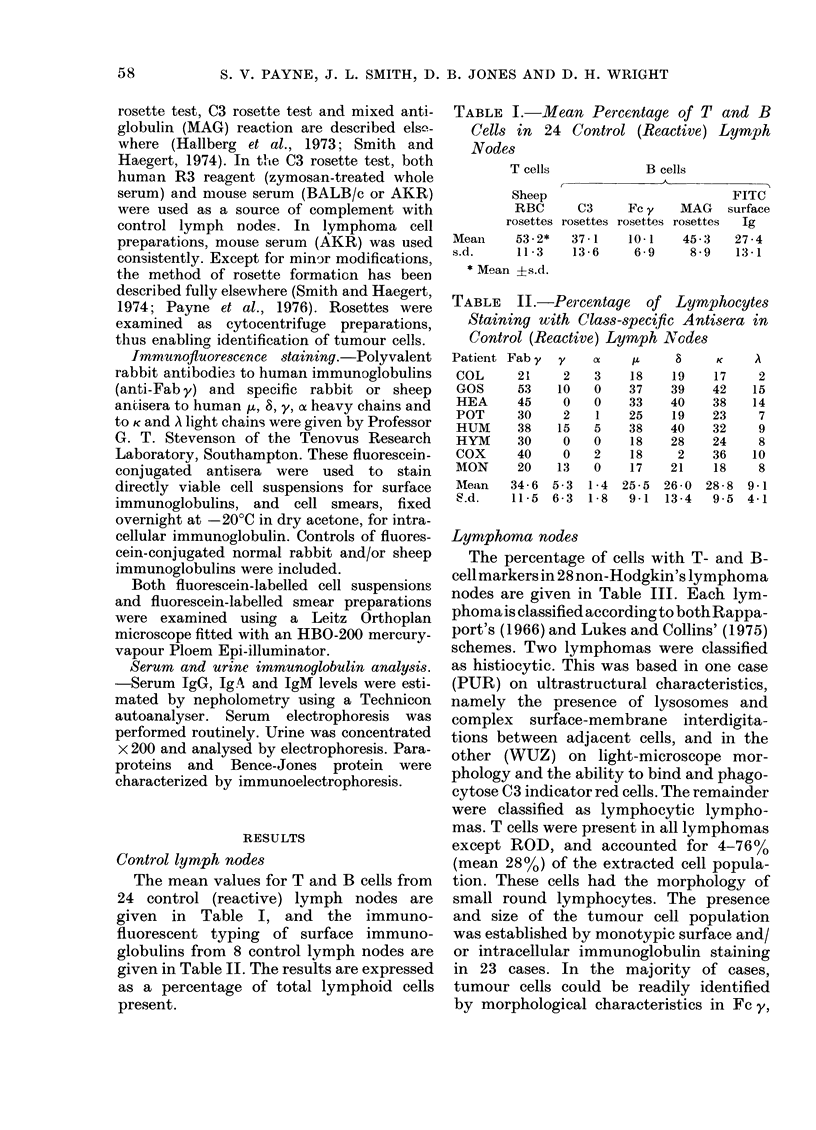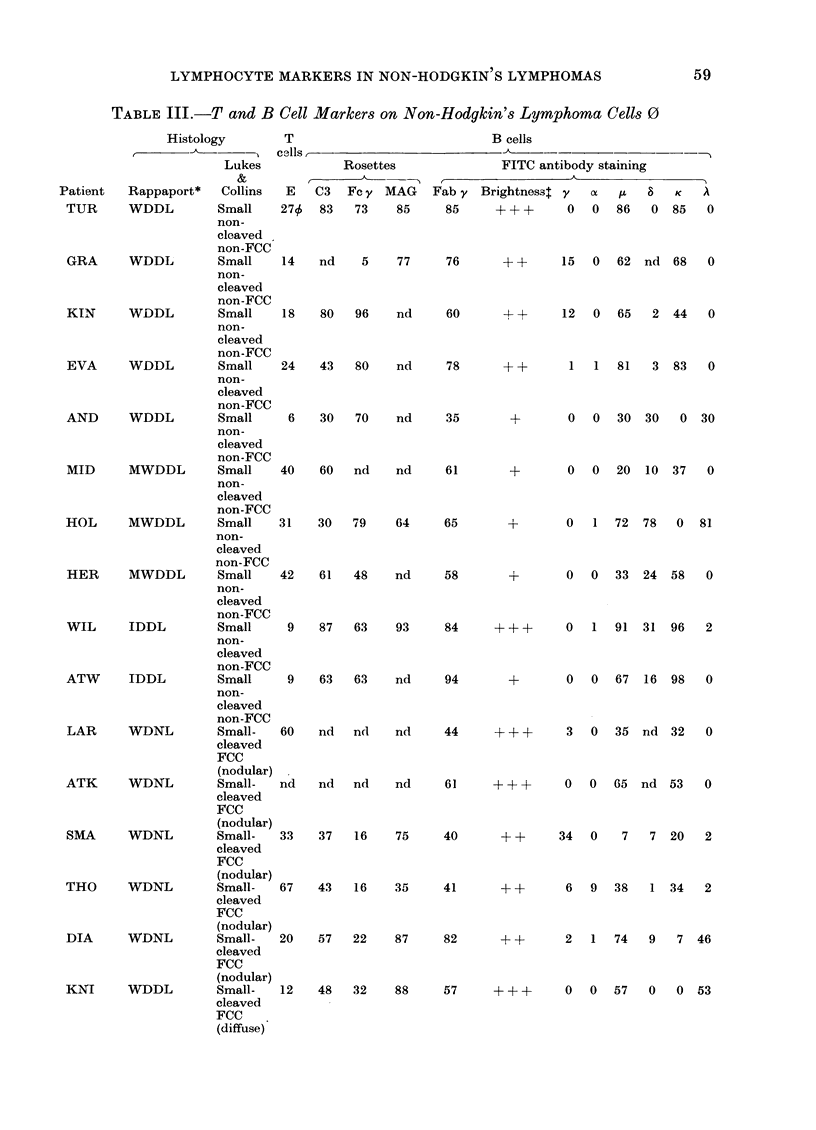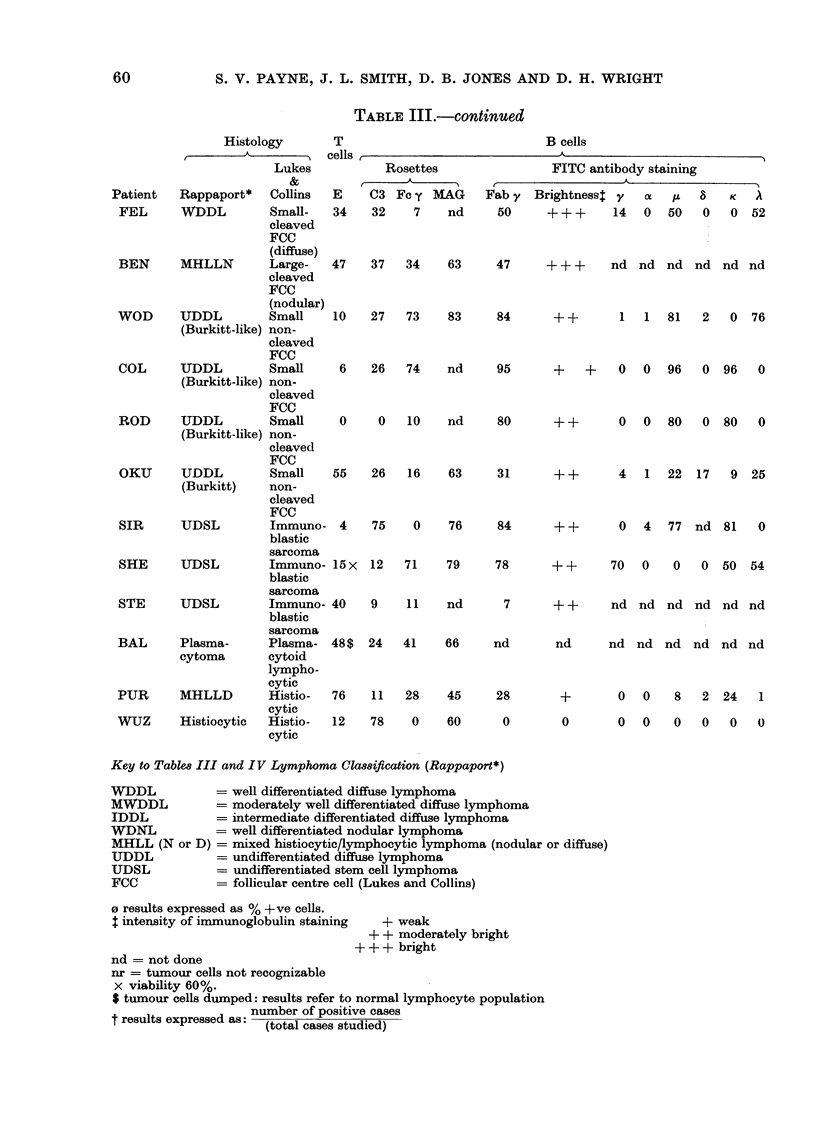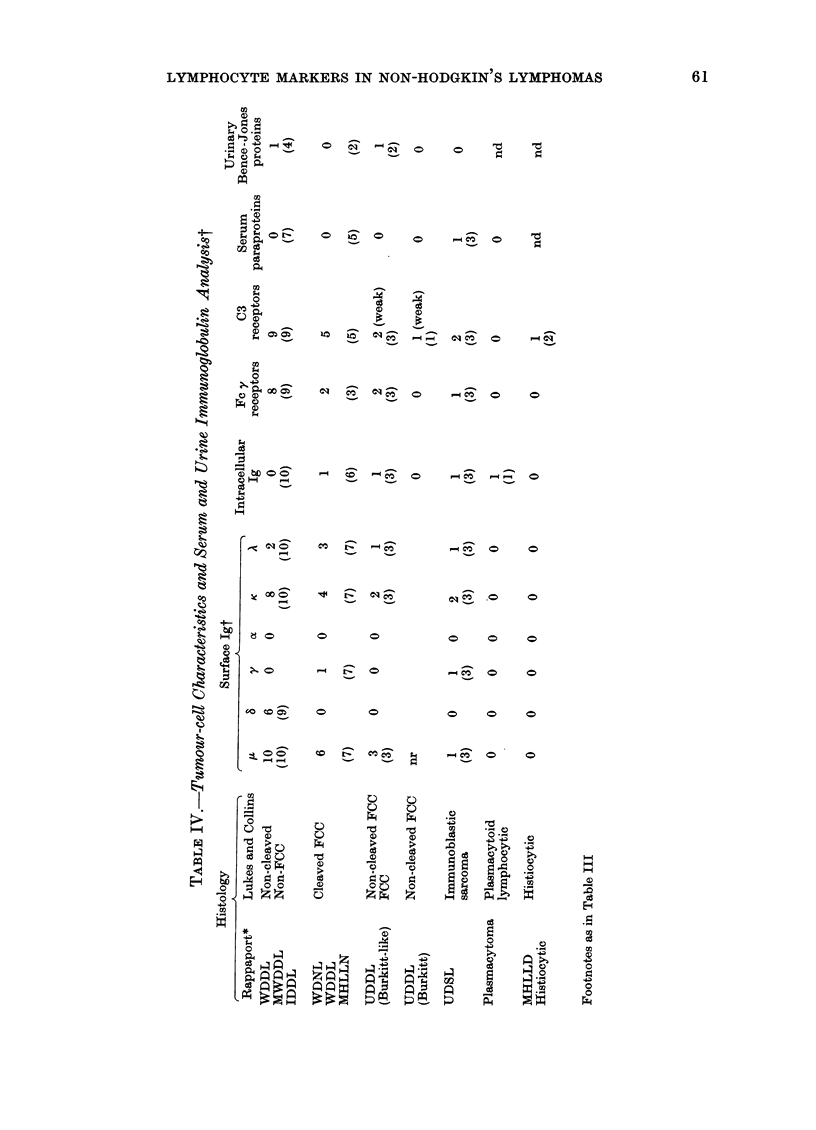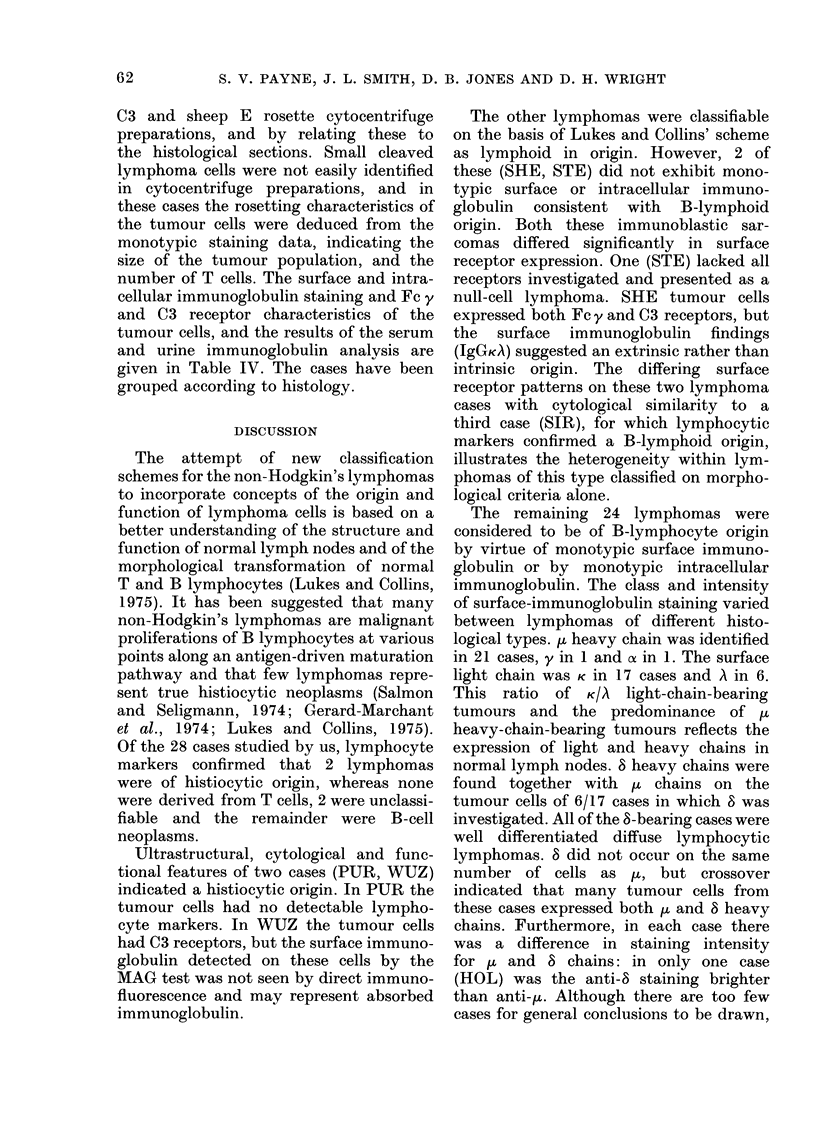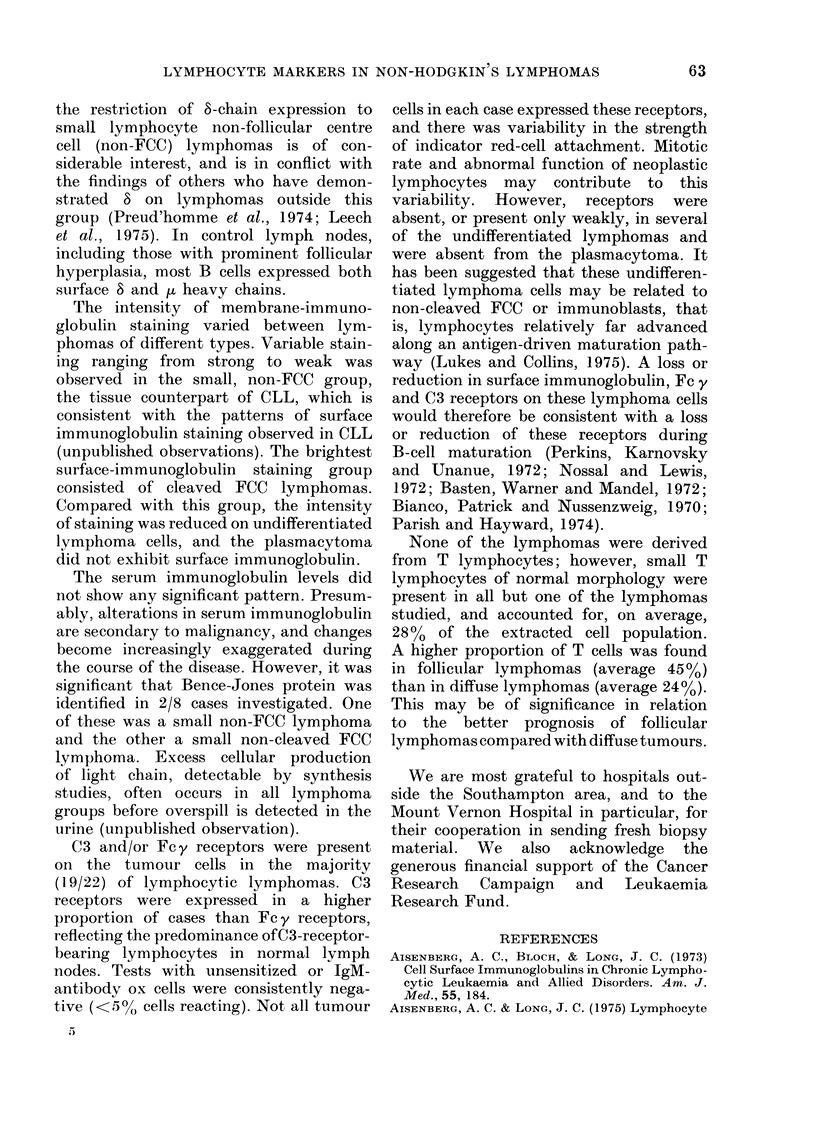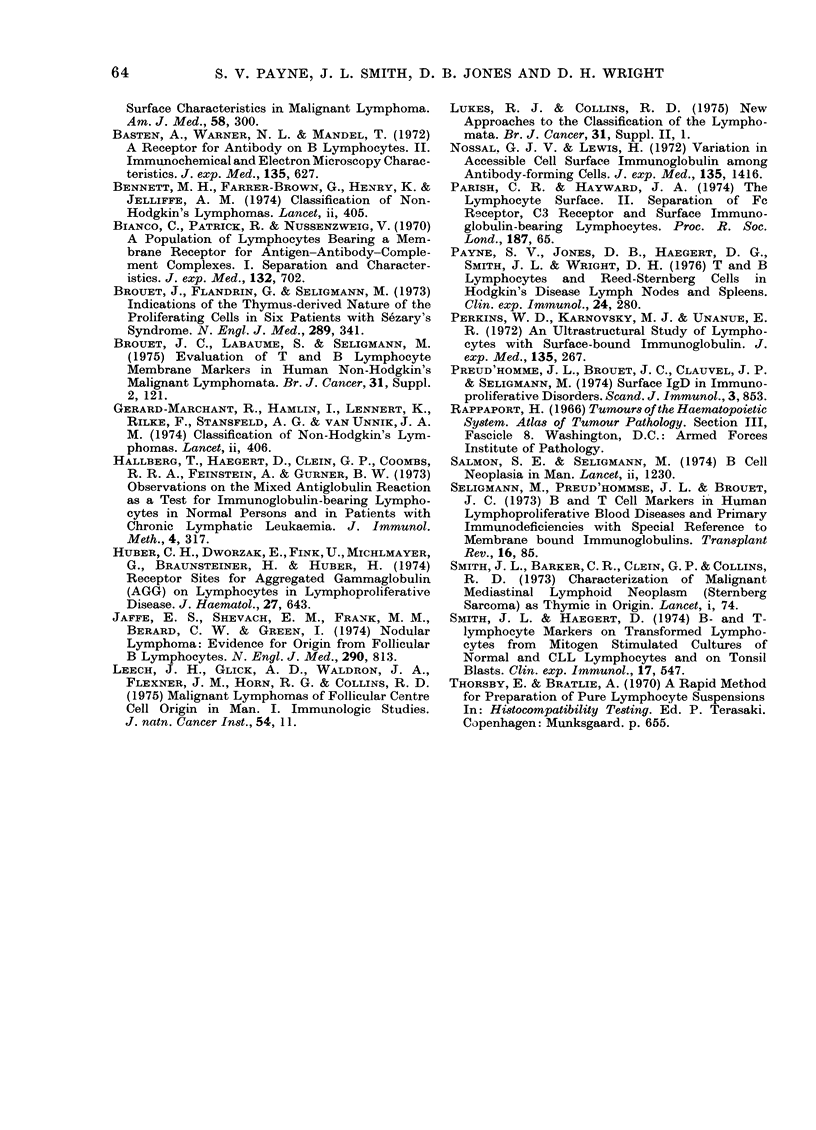# Lymphocyte markers in non-Hodgkin's lymphomas.

**DOI:** 10.1038/bjc.1977.154

**Published:** 1977-07

**Authors:** S. V. Payne, J. L. Smith, D. B. Jones, D. H. Wright

## Abstract

The lymphocyte marker pattern of non-Hodgkin's lymphoma cells was related to current concepts of lymphoma classification. In a series of 28 lymphomas lymphocyte markers indicated that 2 were of histiocytic origin, 2 were unclassifiable, none were derived from T cells and the remainder were B-cell neoplasms. The immunoglobulin heavy chain associated with the B-cell tumours was gamma in one case, alpha in one case but was mu in the majority of cases, reflecting the predominance of this heavy chain, together with delta chains, on normal lymph node lymphocytes in man. delta chains accompanied mu chains on the tumour cells in 6/17 lymphomas in which anti-delta staining was performed. delta chains were not found on any lymphomas other than well differentiated diffuse lymphocytic types. There was evidence of a reduction in surface immunoglobulin, Fcgamma and C3 receptors on undifferentiated lymphoma cells. T lymphocytes of normal morphology were present in all lymphomas except one, and were more numerous in follicular lymphomas than in diffuse tumours.


*Br. J. Cancer* (1977) **36**, 57

### LYMPHOCYTE MARKERS IN NON-HODGKIN'S LYMPHOMAS

S. V. PAYNE, J. L. SMITH,* D. B. JONES AND D. H. WRIGHT

*From the Department of Pathology, Faculty of Medicine, and *Immunology Unit, Tenovus Research Laboratory, Southampton University Hospital*

Received 10 December 1976 Accepted 9 March 1977

**Summary.—The lymphocyte marker pattern of non-Hodgkin's lymphoma cells was related to current concepts of lymphoma classification. In a series of 28 lymphomas, lymphocyte markers indicated that 2 were of histiocytic origin, 2 were unclassifiable, none were derived from T cells and the remainder were B-cell neoplasms. The immunoglobulin heavy chain associated with the B-cell tumours was $\gamma$ in one case, $\alpha$ in one case but was $\mu$ in the majority of cases, reflecting the predominance of this heavy chain, together with $\delta$ chains, on normal lymph node lymphocytes in man. $\delta$ chains accompanied $\mu$ chains on the tumour cells in 6/17 lymphomas in which anti-$\delta$ staining was performed. $\delta$ chains were not found on any lymphomas other than well differentiated diffuse lymphocytic types. There was evidence of a reduction in surface immunoglobulin, Fc$\gamma$ and C3 receptors on undifferentiated lymphoma cells. T lymphocytes of normal morphology were present in all lymphomas except one, and were more numerous in follicular lymphomas than in diffuse tumours.**

MANY lymphoproliferative diseases involving peripheral blood and bone marrow have been shown to originate from distinct lymphocyte populations defined by surface markers for T and B lymphocytes (Seligmann, Preud'homme and Brouet, 1973; Aisenberg, Bloch and Long, 1973; Brouet, Flandrin and Seligmann, 1973). This kind of analysis has more recently been applied to malignant lymphomas, the solid tumours of the immune system (Smith *et al.*, 1973; Jaffe *et al.*, 1974; Huber *et al.*, 1974; Aisenberg and Long, 1975; Leech *et al.*, 1975) Brouet, Labaume and Seligmann, 1975). The classification of these diseases is currently receiving a critical re-analysis in the light of current immunological understanding of the function of the components of the immune system (Gerard-Marchant *et al.*, 1974; Lukes and Collins, 1975; Bennett *et al.*, 1974). We have investigated a group of untreated non-Hodgkin's lymphomas using a panel of lymphocyte markers, including surface and intracellular immunoglobulin, Fc$\gamma$ and C3 receptors and sheep erythrocyte (E) receptors. The receptor pattern of these lymphoma cells has been related to normal lymphocyte maturation and to current concepts of lymphoma classification.

### MATERIALS AND METHODS

*Lymph nodes and peripheral blood.*—Lymphoma tissue was obtained from biopsy specimens taken from 27 untreated patients. One patient (MID) had received treatment. Uninvolved nodes from patients with carcinoma, and nodes showing non-specific reactive changes, were used as controls.

Biopsy tissue was finely minced and teased in cold HEPES-buffered Eagles' medium (HEPES-MEM, Biocult Laboratories, Paisley), filtered through wire gauze and layered over Ficoll–Triosil (Thorsby and Bratlie, 1970). Cells collected at the interface were washed ×3 by centrifugation (150 *g*, 10 min) and the final pellet was resuspended at a density of $2 \times 10^6$ cells/ml in HEPES-MEM with 0·2% bovine serum albumin (BSA). Viability was greater than 80% in all cases except in one undifferentiated stem cell lymphoma (see results).

*Rosette tests.*—Full details of cell preparation for the sheep rosette test (E), Fc$\gamma$

rosette test, C3 rosette test and mixed antiglobulin (MAG) reaction are described elsewhere (Hallberg *et al.*, 1973; Smith and Haegert, 1974). In the C3 rosette test, both human R3 reagent (zymosan-treated whole serum) and mouse serum (BALB/c or AKR) were used as a source of complement with control lymph nodes. In lymphoma cell preparations, mouse serum (AKR) was used consistently. Except for minor modifications, the method of rosette formation has been described fully elsewhere (Smith and Haegert, 1974; Payne *et al.*, 1976). Rosettes were examined as cytocentrifuge preparations, thus enabling identification of tumour cells.

*Immunofluorescence staining.*—Polyvalent rabbit antibodies to human immunoglobulins (anti-Fab $\gamma$) and specific rabbit or sheep antisera to human $\mu$, $\delta$, $\gamma$, $\alpha$ heavy chains and to $\kappa$ and $\lambda$ light chains were given by Professor G. T. Stevenson of the Tenovus Research Laboratory, Southampton. These fluorescein-conjugated antisera were used to stain directly viable cell suspensions for surface immunoglobulins, and cell smears, fixed overnight at —20°C in dry acetone, for intracellular immunoglobulin. Controls of fluorescein-conjugated normal rabbit and/or sheep immunoglobulins were included.

Both fluorescein-labelled cell suspensions and fluorescein-labelled smear preparations were examined using a Leitz Orthoplan microscope fitted with an HBO-200 mercury-vapour Ploem Epi-illuminator.

*Serum and urine immunoglobulin analysis.*—Serum IgG, IgA and IgM levels were estimated by nephelometry using a Technicon autoanalyser. Serum electrophoresis was performed routinely. Urine was concentrated ×200 and analysed by electrophoresis. Paraproteins and Bence-Jones protein were characterized by immunoelectrophoresis.

### RESULTS

### *Control lymph nodes*

The mean values for T and B cells from 24 control (reactive) lymph nodes are given in Table I, and the immunofluorescent typing of surface immunoglobulins from 8 control lymph nodes are given in Table II. The results are expressed as a percentage of total lymphoid cells present.

TABLE I.—*Mean Percentage of T and B Cells in 24 Control (Reactive) Lymph Nodes*

| | T cells | B cells | | | |
|---|---|---|---|---|---|
| | Sheep RBC rosettes | C3 rosettes | Fc $\gamma$ rosettes | MAG rosettes | FITC surface Ig |
| Mean | 53·2* | 37·1 | 10·1 | 45·3 | 27·4 |
| s.d. | 11·3 | 13·6 | 6·9 | 8·9 | 13·1 |

* Mean ±s.d.

TABLE II.—*Percentage of Lymphocytes Staining with Class-specific Antisera in Control (Reactive) Lymph Nodes*

| Patient | Fab $\gamma$ | $\gamma$ | $\alpha$ | $\mu$ | $\delta$ | $\kappa$ | $\lambda$ |
|---|---|---|---|---|---|---|---|
| COL | 21 | 2 | 3 | 18 | 19 | 17 | 2 |
| GOS | 53 | 10 | 0 | 37 | 39 | 42 | 15 |
| HEA | 45 | 0 | 0 | 33 | 40 | 38 | 14 |
| POT | 30 | 2 | 1 | 25 | 19 | 23 | 7 |
| HUM | 38 | 15 | 5 | 38 | 40 | 32 | 9 |
| HYM | 30 | 0 | 0 | 18 | 28 | 24 | 8 |
| COX | 40 | 0 | 2 | 18 | 2 | 36 | 10 |
| MON | 20 | 13 | 0 | 17 | 21 | 18 | 8 |
| Mean | 34·6 | 5·3 | 1·4 | 25·5 | 26·0 | 28·8 | 9·1 |
| S.d. | 11·5 | 6·3 | 1·8 | 9·1 | 13·4 | 9·5 | 4·1 |

### *Lymphoma nodes*

The percentage of cells with T- and B-cell markers in 28 non-Hodgkin's lymphoma nodes are given in Table III. Each lymphoma is classified according to both Rappaport's (1966) and Lukes and Collins' (1975) schemes. Two lymphomas were classified as histiocytic. This was based in one case (PUR) on ultrastructural characteristics, namely the presence of lysosomes and complex surface-membrane interdigitations between adjacent cells, and in the other (WUZ) on light-microscope morphology and the ability to bind and phagocytose C3 indicator red cells. The remainder were classified as lymphocytic lymphomas. T cells were present in all lymphomas except ROD, and accounted for 4–76% (mean 28%) of the extracted cell population. These cells had the morphology of small round lymphocytes. The presence and size of the tumour cell population was established by monotypic surface and/or intracellular immunoglobulin staining in 23 cases. In the majority of cases, tumour cells could be readily identified by morphological characteristics in Fc $\gamma$,

TABLE III.—*T and B Cell Markers on Non-Hodgkin's Lymphoma Cells Ø*

| | Histology | | T cells | B cells | | | | | | | | | | |
|---|---|---|---|---|---|---|---|---|---|---|---|---|---|---|
| | | | | Rosettes | | | FITC antibody staining | | | | | | | |
| Patient | Rappaport* | Lukes & Collins | E | C3 | Fc γ | MAG | Fab γ | Brightness‡ | γ | α | μ | δ | κ | λ |
| TUR | WDDL | Small non-cleaved non-FCC | 27ϕ | 83 | 73 | 85 | 85 | +++ | 0 | 0 | 86 | 0 | 85 | 0 |
| GRA | WDDL | Small non-cleaved non-FCC | 14 | nd | 5 | 77 | 76 | ++ | 15 | 0 | 62 | nd | 68 | 0 |
| KIN | WDDL | Small non-cleaved non-FCC | 18 | 80 | 96 | nd | 60 | ++ | 12 | 0 | 65 | 2 | 44 | 0 |
| EVA | WDDL | Small non-cleaved non-FCC | 24 | 43 | 80 | nd | 78 | ++ | 1 | 1 | 81 | 3 | 83 | 0 |
| AND | WDDL | Small non-cleaved non-FCC | 6 | 30 | 70 | nd | 35 | + | 0 | 0 | 30 | 30 | 0 | 30 |
| MID | MWDDL | Small non-cleaved non-FCC | 40 | 60 | nd | nd | 61 | + | 0 | 0 | 20 | 10 | 37 | 0 |
| HOL | MWDDL | Small non-cleaved non-FCC | 31 | 30 | 79 | 64 | 65 | + | 0 | 1 | 72 | 78 | 0 | 81 |
| HER | MWDDL | Small non-cleaved non-FCC | 42 | 61 | 48 | nd | 58 | + | 0 | 0 | 33 | 24 | 58 | 0 |
| WIL | IDDL | Small non-cleaved non-FCC | 9 | 87 | 63 | 93 | 84 | +++ | 0 | 1 | 91 | 31 | 96 | 2 |
| ATW | IDDL | Small non-cleaved non-FCC | 9 | 63 | 63 | nd | 94 | + | 0 | 0 | 67 | 16 | 98 | 0 |
| LAR | WDNL | Small-cleaved FCC (nodular) | 60 | nd | nd | nd | 44 | +++ | 3 | 0 | 35 | nd | 32 | 0 |
| ATK | WDNL | Small-cleaved FCC (nodular) | nd | nd | nd | nd | 61 | +++ | 0 | 0 | 65 | nd | 53 | 0 |
| SMA | WDNL | Small-cleaved FCC (nodular) | 33 | 37 | 16 | 75 | 40 | ++ | 34 | 0 | 7 | 7 | 20 | 2 |
| THO | WDNL | Small-cleaved FCC (nodular) | 67 | 43 | 16 | 35 | 41 | ++ | 6 | 9 | 38 | 1 | 34 | 2 |
| DIA | WDNL | Small-cleaved FCC (nodular) | 20 | 57 | 22 | 87 | 82 | ++ | 2 | 1 | 74 | 9 | 7 | 46 |
| KNI | WDDL | Small-cleaved FCC (diffuse) | 12 | 48 | 32 | 88 | 57 | +++ | 0 | 0 | 57 | 0 | 0 | 53 |

TABLE III.—*continued*

| | Histology | | T cells | B cells | | | | | | | | | | |
|---|---|---|---|---|---|---|---|---|---|---|---|---|---|---|
| | | | | Rosettes | | | FITC antibody staining | | | | | | | |
| Patient | Rappaport* | Lukes & Collins | E | C3 | Fc γ | MAG | Fab γ | Brightness‡ | γ | α | μ | δ | κ | λ |
| FEL | WDDL | Small-cleaved FCC (diffuse) | 34 | 32 | 7 | nd | 50 | +++ | 14 | 0 | 50 | 0 | 0 | 52 |
| BEN | MHLLN | Large-cleaved FCC (nodular) | 47 | 37 | 34 | 63 | 47 | +++ | nd | nd | nd | nd | nd | nd |
| WOD | UDDL (Burkitt-like) | Small non-cleaved FCC | 10 | 27 | 73 | 83 | 84 | ++ | 1 | 1 | 81 | 2 | 0 | 76 |
| COL | UDDL (Burkitt-like) | Small non-cleaved FCC | 6 | 26 | 74 | nd | 95 | + + | 0 | 0 | 96 | 0 | 96 | 0 |
| ROD | UDDL (Burkitt-like) | Small non-cleaved FCC | 0 | 0 | 10 | nd | 80 | ++ | 0 | 0 | 80 | 0 | 80 | 0 |
| OKU | UDDL (Burkitt) | Small non-cleaved FCC | 55 | 26 | 16 | 63 | 31 | ++ | 4 | 1 | 22 | 17 | 9 | 25 |
| SIR | UDSL | Immuno-blastic sarcoma | 4 | 75 | 0 | 76 | 84 | ++ | 0 | 4 | 77 | nd | 81 | 0 |
| SHE | UDSL | Immuno-blastic sarcoma | 15× | 12 | 71 | 79 | 78 | ++ | 70 | 0 | 0 | 0 | 50 | 54 |
| STE | UDSL | Immuno-blastic sarcoma | 40 | 9 | 11 | nd | 7 | ++ | nd | nd | nd | nd | nd | nd |
| BAL | Plasma-cytoma | Plasma-cytoid lympho-cytic | 48$ | 24 | 41 | 66 | nd | nd | nd | nd | nd | nd | nd | nd |
| PUR | MHLLD | Histio-cytic | 76 | 11 | 28 | 45 | 28 | + | 0 | 0 | 8 | 2 | 24 | 1 |
| WUZ | Histiocytic | Histio-cytic | 12 | 78 | 0 | 60 | 0 | 0 | 0 | 0 | 0 | 0 | 0 | 0 |

*Key to Tables III and IV Lymphoma Classification (Rappaport*)*

WDDL = well differentiated diffuse lymphoma
MWDDL = moderately well differentiated diffuse lymphoma
IDDL = intermediate differentiated diffuse lymphoma
WDNL = well differentiated nodular lymphoma
MHLL (N or D) = mixed histiocytic/lymphocytic lymphoma (nodular or diffuse)
UDDL = undifferentiated diffuse lymphoma
UDSL = undifferentiated stem cell lymphoma
FCC = follicular centre cell (Lukes and Collins)

ø results expressed as % +ve cells.
‡ intensity of immunoglobulin staining + weak
++ moderately bright
+++ bright
nd = not done
nr = tumour cells not recognizable
× viability 60%.
$ tumour cells dumped: results refer to normal lymphocyte population
† results expressed as: $\frac{\text{number of positive cases}}{\text{(total cases studied)}}$

TABLE IV.—*Tumour-cell Characteristics and Serum and Urine Immunoglobulin Analysis*†

| Histology | | Surface Ig† | | | | | | Intracellular Ig | Fc $\gamma$ receptors | C3 receptors | Serum paraproteins | Urinary Bence-Jones proteins |
|---|---|---|---|---|---|---|---|---|---|---|---|---|
| Rappaport* | Lukes and Collins | $\mu$ | $\delta$ | $\gamma$ | $\alpha$ | $\kappa$ | $\lambda$ | | | | | |
| WDDL<br>MWDDL<br>IDDL | Non-cleaved<br>Non-FCC | 10<br>(10) | 6<br>(9) | 0 | 0 | 8<br>(10) | 2<br>(10) | 0<br>(10) | 8<br>(9) | 9<br>(9) | 0<br>(7) | 1<br>(4) |
| WDNL<br>WDDL<br>MHLLN | Cleaved FCC | 6<br>(7) | 0 | 1<br>(7) | 0 | 4<br>(7) | 3<br>(7) | 1<br>(6) | 2<br>(3) | 5<br>(5) | 0<br>(5) | 0<br>(2) |
| UDDL<br>(Burkitt-like) | Non-cleaved FCC<br>FCC | 3<br>(3) | 0 | 0 | 0 | 2<br>(3) | 1<br>(3) | 1<br>(3) | 2<br>(3) | 2 (weak)<br>(3) | 0 | 1<br>(2) |
| UDDL<br>(Burkitt) | Non-cleaved FCC | nr | | | | | | 0 | 0 | 1 (weak)<br>(1) | 0 | 0 |
| UDSL | Immunoblastic<br>sarcoma | 1<br>(3) | 0 | 1<br>(3) | 0 | 2<br>(3) | 1<br>(3) | 1<br>(3) | 1<br>(3) | 2<br>(3) | 1<br>(3) | 0 |
| Plasmacytoma | Plasmacytoid<br>lymphocytic | 0 | 0 | 0 | 0 | 0 | 0 | 1<br>(1) | 0 | 0 | 0 | nd |
| MHLLD<br>Histiocytic | Histiocytic | 0 | 0 | 0 | 0 | 0 | 0 | 0 | 0 | 1<br>(2) | nd | nd |

Footnotes as in Table III

C3 and sheep E rosette cytocentrifuge preparations, and by relating these to the histological sections. Small cleaved lymphoma cells were not easily identified in cytocentrifuge preparations, and in these cases the rosetting characteristics of the tumour cells were deduced from the monotypic staining data, indicating the size of the tumour population, and the number of T cells. The surface and intracellular immunoglobulin staining and Fc $\gamma$ and C3 receptor characteristics of the tumour cells, and the results of the serum and urine immunoglobulin analysis are given in Table IV. The cases have been grouped according to histology.

### DISCUSSION

The attempt of new classification schemes for the non-Hodgkin's lymphomas to incorporate concepts of the origin and function of lymphoma cells is based on a better understanding of the structure and function of normal lymph nodes and of the morphological transformation of normal T and B lymphocytes (Lukes and Collins, 1975). It has been suggested that many non-Hodgkin's lymphomas are malignant proliferations of B lymphocytes at various points along an antigen-driven maturation pathway and that few lymphomas represent true histiocytic neoplasms (Salmon and Seligmann, 1974; Gerard-Marchant *et al.*, 1974; Lukes and Collins, 1975). Of the 28 cases studied by us, lymphocyte markers confirmed that 2 lymphomas were of histiocytic origin, whereas none were derived from T cells, 2 were unclassifiable and the remainder were B-cell neoplasms.

Ultrastructural, cytological and functional features of two cases (PUR, WUZ) indicated a histiocytic origin. In PUR the tumour cells had no detectable lymphocyte markers. In WUZ the tumour cells had C3 receptors, but the surface immunoglobulin detected on these cells by the MAG test was not seen by direct immunofluorescence and may represent absorbed immunoglobulin.

The other lymphomas were classifiable on the basis of Lukes and Collins' scheme as lymphoid in origin. However, 2 of these (SHE, STE) did not exhibit monotypic surface or intracellular immunoglobulin consistent with B-lymphoid origin. Both these immunoblastic sarcomas differed significantly in surface receptor expression. One (STE) lacked all receptors investigated and presented as a null-cell lymphoma. SHE tumour cells expressed both Fc $\gamma$ and C3 receptors, but the surface immunoglobulin findings (IgG$\kappa\lambda$) suggested an extrinsic rather than intrinsic origin. The differing surface receptor patterns on these two lymphoma cases with cytological similarity to a third case (SIR), for which lymphocytic markers confirmed a B-lymphoid origin, illustrates the heterogeneity within lymphomas of this type classified on morphological criteria alone.

The remaining 24 lymphomas were considered to be of B-lymphocyte origin by virtue of monotypic surface immunoglobulin or by monotypic intracellular immunoglobulin. The class and intensity of surface-immunoglobulin staining varied between lymphomas of different histological types. $\mu$ heavy chain was identified in 21 cases, $\gamma$ in 1 and $\alpha$ in 1. The surface light chain was $\kappa$ in 17 cases and $\lambda$ in 6. This ratio of $\kappa/\lambda$ light-chain-bearing tumours and the predominance of $\mu$ heavy-chain-bearing tumours reflects the expression of light and heavy chains in normal lymph nodes. $\delta$ heavy chains were found together with $\mu$ chains on the tumour cells of 6/17 cases in which $\delta$ was investigated. All of the $\delta$-bearing cases were well differentiated diffuse lymphocytic lymphomas. $\delta$ did not occur on the same number of cells as $\mu$, but crossover indicated that many tumour cells from these cases expressed both $\mu$ and $\delta$ heavy chains. Furthermore, in each case there was a difference in staining intensity for $\mu$ and $\delta$ chains: in only one case (HOL) was the anti-$\delta$ staining brighter than anti-$\mu$. Although there are too few cases for general conclusions to be drawn, the restriction of δ-chain expression to small lymphocyte non-follicular centre cell (non-FCC) lymphomas is of considerable interest, and is in conflict with the findings of others who have demonstrated δ on lymphomas outside this group (Preud'homme *et al.*, 1974; Leech *et al.*, 1975). In control lymph nodes, including those with prominent follicular hyperplasia, most B cells expressed both surface δ and μ heavy chains.

The intensity of membrane-immunoglobulin staining varied between lymphomas of different types. Variable staining ranging from strong to weak was observed in the small, non-FCC group, the tissue counterpart of CLL, which is consistent with the patterns of surface immunoglobulin staining observed in CLL (unpublished observations). The brightest surface-immunoglobulin staining group consisted of cleaved FCC lymphomas. Compared with this group, the intensity of staining was reduced on undifferentiated lymphoma cells, and the plasmacytoma did not exhibit surface immunoglobulin.

The serum immunoglobulin levels did not show any significant pattern. Presumably, alterations in serum immunoglobulin are secondary to malignancy, and changes become increasingly exaggerated during the course of the disease. However, it was significant that Bence-Jones protein was identified in 2/8 cases investigated. One of these was a small non-FCC lymphoma and the other a small non-cleaved FCC lymphoma. Excess cellular production of light chain, detectable by synthesis studies, often occurs in all lymphoma groups before overspill is detected in the urine (unpublished observation).

C3 and/or Fcγ receptors were present on the tumour cells in the majority (19/22) of lymphocytic lymphomas. C3 receptors were expressed in a higher proportion of cases than Fcγ receptors, reflecting the predominance of C3-receptor-bearing lymphocytes in normal lymph nodes. Tests with unsensitized or IgM-antibody ox cells were consistently negative ($<5\%$ cells reacting). Not all tumour cells in each case expressed these receptors, and there was variability in the strength of indicator red-cell attachment. Mitotic rate and abnormal function of neoplastic lymphocytes may contribute to this variability. However, receptors were absent, or present only weakly, in several of the undifferentiated lymphomas and were absent from the plasmacytoma. It has been suggested that these undifferentiated lymphoma cells may be related to non-cleaved FCC or immunoblasts, that is, lymphocytes relatively far advanced along an antigen-driven maturation pathway (Lukes and Collins, 1975). A loss or reduction in surface immunoglobulin, Fc γ and C3 receptors on these lymphoma cells would therefore be consistent with a loss or reduction of these receptors during B-cell maturation (Perkins, Karnovsky and Unanue, 1972; Nossal and Lewis, 1972; Basten, Warner and Mandel, 1972; Bianco, Patrick and Nussenzweig, 1970; Parish and Hayward, 1974).

None of the lymphomas were derived from T lymphocytes; however, small T lymphocytes of normal morphology were present in all but one of the lymphomas studied, and accounted for, on average, 28% of the extracted cell population. A higher proportion of T cells was found in follicular lymphomas (average 45%) than in diffuse lymphomas (average 24%). This may be of significance in relation to the better prognosis of follicular lymphomas compared with diffuse tumours.

We are most grateful to hospitals outside the Southampton area, and to the Mount Vernon Hospital in particular, for their cooperation in sending fresh biopsy material. We also acknowledge the generous financial support of the Cancer Research Campaign and Leukaemia Research Fund.

### REFERENCES

AISENBERG, A. C., BLOCH, & LONG, J. C. (1973) Cell Surface Immunoglobulins in Chronic Lymphocytic Leukaemia and Allied Disorders. *Am. J. Med.*, **55**, 184.

AISENBERG, A. C. & LONG, J. C. (1975) Lymphocyte

Surface Characteristics in Malignant Lymphoma. *Am. J. Med.*, **58**, 300.

BASTEN, A., WARNER, N. L. & MANDEL, T. (1972) A Receptor for Antibody on B Lymphocytes. II. Immunochemical and Electron Microscopy Characteristics. *J. exp. Med.*, **135**, 627.

BENNETT, M. H., FARRER-BROWN, G., HENRY, K. & JELLIFFE, A. M. (1974) Classification of Non-Hodgkin's Lymphomas. *Lancet*, ii, 405.

BIANCO, C., PATRICK, R. & NUSSENZWEIG, V. (1970) A Population of Lymphocytes Bearing a Membrane Receptor for Antigen–Antibody–Complement Complexes. I. Separation and Characteristics. *J. exp. Med.*, **132**, 702.

BROUET, J., FLANDRIN, G. & SELIGMANN, M. (1973) Indications of the Thymus-derived Nature of the Proliferating Cells in Six Patients with Sézary's Syndrome. *N. Engl. J. Med.*, **289**, 341.

BROUET, J. C., LABAUME, S. & SELIGMANN, M. (1975) Evaluation of T and B Lymphocyte Membrane Markers in Human Non-Hodgkin's Malignant Lymphomata. *Br. J. Cancer*, **31**, Suppl. 2, 121.

GERARD-MARCHANT, R., HAMLIN, I., LENNERT, K., RILKE, F., STANSFELD, A. G. & VAN UNNIK, J. A. M. (1974) Classification of Non-Hodgkin's Lymphomas. *Lancet*, ii, 406.

HALLBERG, T., HAEGERT, D., CLEIN, G. P., COOMBS, R. R. A., FEINSTEIN, A. & GURNER, B. W. (1973) Observations on the Mixed Antiglobulin Reaction as a Test for Immunoglobulin-bearing Lymphocytes in Normal Persons and in Patients with Chronic Lymphatic Leukaemia. *J. Immunol. Meth.*, **4**, 317.

HUBER, C. H., DWORZAK, E., FINK, U., MICHLMAYER, G., BRAUNSTEINER, H. & HUBER, H. (1974) Receptor Sites for Aggregated Gammaglobulin (AGG) on Lymphocytes in Lymphoproliferative Disease. *J. Haematol.*, **27**, 643.

JAFFE, E. S., SHEVACH, E. M., FRANK, M. M., BERARD, C. W. & GREEN, I. (1974) Nodular Lymphoma: Evidence for Origin from Follicular B Lymphocytes. *N. Engl. J. Med.*, **290**, 813.

LEECH, J. H., GLICK, A. D., WALDRON, J. A., FLEXNER, J. M., HORN, R. G. & COLLINS, R. D. (1975) Malignant Lymphomas of Follicular Centre Cell Origin in Man. I. Immunologic Studies. *J. natn. Cancer Inst.*, **54**, 11.

LUKES, R. J. & COLLINS, R. D. (1975) New Approaches to the Classification of the Lymphomata. *Br. J. Cancer*, **31**, Suppl. II, 1.

NOSSAL, G. J. V. & LEWIS, H. (1972) Variation in Accessible Cell Surface Immunoglobulin among Antibody-forming Cells. *J. exp. Med.*, **135**, 1416.

PARISH, C. R. & HAYWARD, J. A. (1974) The Lymphocyte Surface. II. Separation of Fc Receptor, C3 Receptor and Surface Immunoglobulin-bearing Lymphocytes. *Proc. R. Soc. Lond.*, **187**, 65.

PAYNE, S. V., JONES, D. B., HAEGERT, D. G., SMITH, J. L. & WRIGHT, D. H. (1976) T and B Lymphocytes and Reed-Sternberg Cells in Hodgkin's Disease Lymph Nodes and Spleens. *Clin. exp. Immunol.*, **24**, 280.

PERKINS, W. D., KARNOVSKY, M. J. & UNANUE, E. R. (1972) An Ultrastructural Study of Lymphocytes with Surface-bound Immunoglobulin. *J. exp. Med.*, **135**, 267.

PREUD'HOMME, J. L., BROUET, J. C., CLAUVEL, J. P. & SELIGMANN, M. (1974) Surface IgD in Immunoproliferative Disorders. *Scand. J. Immunol.*, **3**, 853.

RAPPAPORT, H. (1966) *Tumours of the Haematopoietic System. Atlas of Tumour Pathology.* Section III, Fascicle 8. Washington, D.C.: Armed Forces Institute of Pathology.

SALMON, S. E. & SELIGMANN, M. (1974) B Cell Neoplasia in Man. *Lancet*, ii, 1230.

SELIGMANN, M., PREUD'HOMMSE, J. L. & BROUET, J. C. (1973) B and T Cell Markers in Human Lymphoproliferative Blood Diseases and Primary Immunodeficiencies with Special Reference to Membrane bound Immunoglobulins. *Transplant Rev.*, **16**, 85.

SMITH, J. L., BARKER, C. R., CLEIN, G. P. & COLLINS, R. D. (1973) Characterization of Malignant Mediastinal Lymphoid Neoplasm (Sternberg Sarcoma) as Thymic in Origin. *Lancet*, i, 74.

SMITH, J. L. & HAEGERT, D. (1974) B- and T-lymphocyte Markers on Transformed Lymphocytes from Mitogen Stimulated Cultures of Normal and CLL Lymphocytes and on Tonsil Blasts. *Clin. exp. Immunol.*, **17**, 547.

THORSBY, E. & BRATLIE, A. (1970) A Rapid Method for Preparation of Pure Lymphocyte Suspensions In: *Histocompatibility Testing.* Ed. P. Terasaki. Copenhagen: Munksgaard. p. 655.